# Oxytocin Facilitates Empathic- and Self-embarrassment Ratings by Attenuating Amygdala and Anterior Insula Responses

**DOI:** 10.3389/fendo.2018.00572

**Published:** 2018-09-25

**Authors:** YaYuan Geng, Weihua Zhao, Feng Zhou, Xiaole Ma, Shuxia Yao, Benjamin Becker, Keith M. Kendrick

**Affiliations:** MOE Key Laboratory for Neuroinformation, Clinical Hospital of Chengdu Brain Science Institute, University of Electronic Science and Technology of China, Chengdu, China

**Keywords:** oxytocin, empathy, embarrassment, anxiety, insula, amygdala, prefrontal cortex, sex differences

## Abstract

The hypothalamic neuropeptide oxytocin has been reported to enhance emotional empathy in association with reduced amygdala activation, although to date studies have not investigated empathy for individuals expressing self-conscious, moral emotions which engage mentalizing as well as emotion processing networks. In the current randomized, double-blind placebo controlled functional MRI experiment in 70 male and female subjects we have therefore investigated the effects of intranasal oxytocin (40 IU) on behavioral and neural responses to embarrassment experienced by others or by self. Results showed that oxytocin significantly increased ratings of both empathic and self-embarrassment and concomitantly decreased skin conductance response, activation in the right amygdala and insula but not in the medial prefrontal cortex. The amygdala effects of oxytocin were associated with the magnitude of the skin conductance response and trait anxiety scores. Overall our results demonstrate that oxytocin increases ratings of self- and other embarrassment and that this is associated with reduced physiological arousal and activity in neural circuits involved in emotional arousal. The neural effects of oxytocin were more pronounced stronger in individuals with high trait anxiety suggesting that it may particularly reduce their anxiety in embarrassing situations.

## Introduction

Our ability to empathize with others is a core feature influencing our social behavior through allowing us to understand both what others are thinking and feeling and thereby promoting our social interactions with them. As such, impaired ability to empathize with others is often a core feature of disorders where social communication and interactions are dysfunctional, such as autism spectrum disorders (1), depression (2) and psychopathy (3).

Even though empathy has been extensively investigated, its sub-components and associated neural mechanisms are still not fully established (4). The most prevalent view considers empathy as a multidimensional construct including both cognitive (identifying emotions expressed by another person) and emotional components (being aroused by or feeling the same emotion expressed by another person) (5). Meta-analytic data has suggested that a network including left orbital frontal cortex, left anterior mid-cingulate cortex, left anterior insula and left dorsal medial thalamus is involved in cognitive-evaluative empathy, whereas another network including the dorsal anterior cingulate cortex, bilateral anterior insula, right dorsal medial thalamus and midbrain is involved in affective-perceptual empathy (6). Studies that directly compared the two empathy components additionally proposed that the inferior frontal gyrus is essential for emotional empathy, whereas the superior and middle frontal gyrus and the orbital gyrus are specifically engaged in cognitive empathy (7). However, the derived empathy network can differ dependent upon the paradigm used (4) thus making it important to establish a core network that is maintained across different paradigms.

The hypothalamic neuropeptide oxytocin (OXT) has been implicated in a number of crucial aspects of social cognition and emotional bonds (8, 9) and importantly studies have reported that it can enhance components of empathy (10–12). The most consistent findings have been in studies using paradigms that distinguish cognitive from emotional empathy components. For example, in male Caucasian subjects OXT was found to enhance both direct and indirect aspects of emotional empathy, but not cognitive empathy *per se*, in the Multifaceted Empathy Task (MET) (12). Urbach-Wiethe disorder patients with selective and complete bilateral amygdala-lesions also exhibited deficits in emotional but not cognitive empathy in the MET suggesting that the emotional empathy enhancing effects of OXT might be mediated by the amygdala (12). We recently confirmed these behavioral findings in Chinese male, as well as female subjects using a Chinese version of the MET and demonstrated that OXT effects on emotional empathy were associated with decreased amygdala activity and increased physiological arousal (10). Finally, another study using dynamic, empathy-inducing video clips in which protagonists expressed sadness, happiness, pain or fear demonstrated that OXT exerted no effects on cognitive empathy but selectively enhanced emotional empathy for fear (11). Thus, OXT may particularly enhance emotional empathy, although in the context of the strong modulatory influence of personal and social contextual factors on the specific effects of OXT (8, 13) it is important to extend these observations using other paradigms and contexts where empathic responses are evoked.

Some initial support for OXT also influencing aspects of cognitive empathy has been reported using the reading the mind in the eyes test (RMET) where participants identify from visual cues restricted to the eye regions which of four different complex emotions is being experienced by subjects. Thus, in one study OXT was reported to increase accuracy, particularly for more difficult items (14). However, subsequent studies found that OXT either only increased RMET accuracy for difficult items in individuals with low empathy scores (15) or generally only in individuals with lower socially proficiency associated with higher levels of maternal love withdrawal (16). Furthermore, a final comprehensive study failed to observe any effects of OXT on RMET performance even taking into account item difficulty, gender and valence and subject traits (17). Thus, evidence for OXT enhancing cognitive empathy using the RMET paradigm remains equivocal.

In the current double-blind, placebo-controlled study we therefore investigated whether OXT would enhance empathy in social situations involving more complex moral, “self-conscious,” emotions such as embarrassment. Embarrassment is primarily a self-reflection and self-evaluative process which serves as a way to help humans adapt their behavior to social norms by punishing non-compliance to such norms with a negative emotional state (18). The neural pathways involved in embarrassment include both those controlling mentalizing and emotional arousal (19). Oxytocin has previously been shown to modulate activity in both of these networks in the context of processing self-referential information (mPFC–(20, 21)) and emotional stimuli [amygdala and anterior insula–(22–27)].

We can experience empathic-embarrassment (EE) toward others we see in embarrassing situations irrespective of whether they are familiar or not (28), and so in the current paradigm we investigated OXT effects on behavioral and neural responses to pictures depicting others in embarrassing situations. Additionally, we also asked subjects to imagine their own feelings if they experienced a similar situation themselves (i.e., self-embarrassment–SE). We reasoned that the latter self-related context would involve a stronger mentalizing component, although both contexts should have strong arousal components. On the behavioral level, we hypothesized that, in line with its enhancement of emotional empathy (10, 12), OXT would increase both EE and SE ratings. At the neural level we hypothesized that in line with previous findings, both in the context of emotional empathy (10) and responses to fear and other arousing stimuli (8, 24), OXT would decrease amygdala activity and also change that of the insula (22, 25–27). We additionally hypothesized that given the mentalizing component associated with embarrassment there would be involvement of the mPFC which could also be modulated by OXT given previous findings (20, 21), but in view of the greater self-orientation and mentalizing component in SE we additionally hypothesized that the effects of OXT on mPFC activation would be greater in this condition. We have previously shown that some behavioral and neural effects of OXT on empathy and other behaviors are associated with increased physiological arousal and modulated by autistic traits (10, 29). The neural effects of embarrassment are also modulated by anxiety (30) and autism (19). Thus, in the current study we also investigated the effects of OXT on physiological arousal as assessed by electrodermal activity and associations of its effects with autism and anxiety traits.

## Materials and methods

### Participants

A total of *n* = 70 participants were enrolled in the present randomized double-blind, placebo-controlled between-subject experiment. Participants were randomly assigned to receive OXT (40 International Units, IU) or placebo (PLC) intranasal treatment resulting in 35 participants (female *n* = 17) in the OXT treatment group and 35 (female *n* = 15) in the PLC treatment group. Both groups were of comparable age [mean ± STD, OXT: 22.03 ± 2.15; PLC: 21.86 ± 1.97; *p* = 0.73,*t*_(70)_ = −0.35], education [OXT: 16.09 ± 1.65; PLC: 15.89 ± 1.64; *p* = 0.61, *t*_(70)_ = −0.51], and gender distribution [χ^2^(1) = 0.23, *p* = 0.63]. All participants reported having no past / current physical, psychiatric or neurological disorders or regular/current use of medication or tobacco. Subjects were required to refrain from nicotine, alcohol or caffeine intake for at least 12 h before the experiment. None of the female participants were taking oral contraceptives or were in their menstrual period. The distribution of females estimated to be in their follicular or luteal phases did not differ significantly between the groups (χ^2^ = 0.06, *p* = 1.00). Written informed consent was obtained from each participant before the experiment. The study was approved by the ethical committee of the University of Electronic Science and Technology of China, and all procedures were in accordance with the latest revision of the declaration of Helsinki.

### Experimental paradigm

**Figure 1:**
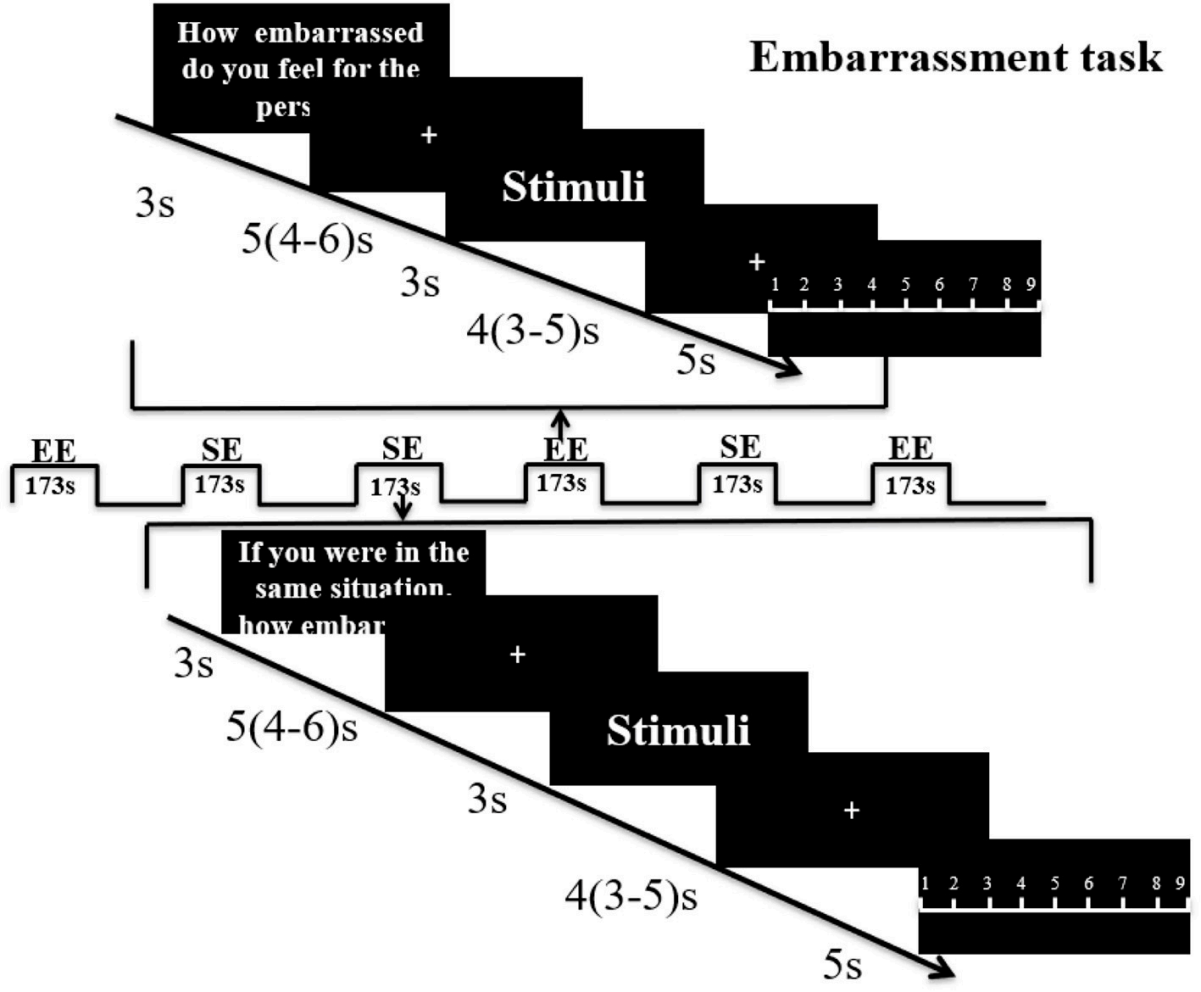
Paradigm for the embarrassment task. Subjects were first shown an instruction for 3 s to indicate whether it was an empathic embarrassment (EE) or self-embarrassment (SE) trial followed by a jittered fixation cross (5 s mean duration with 4–6 s range. The picture stimulus was then presented for 3 s, followed by another fixation jittered mean duration of 4 s (range 3–5 s). A rating slide (1–9) was then shown for 5 s. Each run included one EE block and one SE block of 10 stimuli and the run order was counterbalanced using an ABBA design.

40 pictures depicting male or female protagonists (Chinese protagonists; 20 male, 20 female) in everyday embarrassing situations were evaluated by an independent sample (*n* = 29, 14 females) prior to the experiment to balance mean ratings for empathic embarrassment (EE–how embarrassed do you feel for the person in the picture?) and self-embarrassment (SE–if you were in the same situation, how embarrassed would you feel?) in response to the pictures and the gender of the protagonist (EE: females, 6.54 ± 1.14, males, 6.65 ± 0.89; SE: females, 7.91 ± 0.55, males, 7.58 ± 0.67). No significant differences were found with regard to both EE and SE ratings between male and female participants (EE, *p* = 0.77, *t* = −0.30; SE, *p* = 0.17, *t* = 1.41). For the fMRI experiment stimuli were presented in a mixed block/event related design during four subsequent runs. Each run containing one block of EE and one block of SE trials, with 10 trials of stimuli presented during each block. Each block started with a 3 s cue presentation indicating whether the subject was required to rate EE or SE. Within each block stimuli were presented for 3 s, followed by a jittered low-level baseline during which a fixation cross was presented for 4 s (3–5 s). After each stimulus, subjects were given 5 s to rate EE or SE using a 1–9 Likert rating scale (1 = not at all, 9 = very strong) followed by another 5 s (4–6 s) jittered inter-trial interval. An ABBA block-design was used to counterbalance the order of conditions (see Figure 1 for paradigm details).

### Procedure

To control for potential confounders, all participants completed a test battery of Chinese versions of mood and trait questionnaires before intranasal administration: Beck's Depression Inventory (BDI) (31), Emotional Intelligence Scale (Wleis-C) (32), Empathy Quotient (EQ) (33), Liebowitz Social Anxiety Scale (LSAS) (34) and Positive and Negative Affect Scale (PANAS) (35). Based on previous research reporting that individual variations in autism and anxiety influence (1) embarrassment-associated neural activity (30), as well as (2) effects of OXT on embarrassment-related functional domains (e.g., emotional arousal, empathy and self-appraisal) and neural activity in embarrassment-related regions, including the insula (10, 29, 36–38), levels of anxiety and autism were assessed in the present sample. To this end, participants additionally completed the State Trait Anxiety Inventory (STAI) (39) and Autism Spectrum Quotient (ASQ) (40). Next, subjects self-administered intranasal spray (either 40IU of OXT or PLC lacking the neuropeptide, both supplied by the Sichuan Meike Pharmaceutical Co., Ltd, Sichuan, China). The PLC spray had identical packing and ingredients as the OXT spray (sodium chloride and glycerine, minus the peptide). In accordance with previous recommendations for the intranasal administration of OXT in humans (8, 41), the experimental paradigm started 45 min after treatment. In post-experiment interviews participants were unable to guess better than chance which treatment they had received (34 subjects guessed correctly; χ^2^ = 0.22, *p* = 0.64), confirming successful blinding.

During the experiment electrodermal activity was also measured to assess skin conductance responses (SCR) to the stimuli as an index of autonomic sympathetic activity (42) using the same approach as previously described (10). For the SCR data an event-related analysis approach was employed focusing on SCR responses associated with the presentation of SE and EE stimuli (procedures for preprocessing and event-related analysis of the SCR data were identical to our previous study, details provided in Geng et al. (10).

### *fMRI* acquisition

MRI data was acquired using a GE (General Electric Medical System, Milwaukee, WI, USA) 3T Discovery 750 MRI system with a standard head coil. fMRI time series were acquired using a T2^*^-weighted echo planar imaging pulse sequence (repetition time, 2,000 ms; echo time, 30 ms; slices, 39; thickness, 3.4 mm; gap, 0.6 mm; field of view, 240 × 240 mm^2^; resolution, 64 × 64; flip angle, 90°). Additionally, a high resolution T1-weighted structural image was acquired using a 3D spoiled gradient recalled (SPGR) sequence (repetition time, 6 ms; echo time, 2 ms; flip angle 9°; field of view, 256 × 256 mm^2^; acquisition matrix, 256 × 256; thickness, 1 mm without gap) to exclude subjects with apparent brain pathologies and to improve normalization of the fMRI data.

### *fMRI* data processing

fMRI data were analyzed using SPM12 (Wellcome Trust Center of Neuroimaging, University College London, London, United Kingdom). The first five volumes were discarded from further analyses and images were realigned to the first image to correct for head motion using a six-parameter rigid body algorithm and unwarping. Tissue segmentation, bias-correction and skull-stripping were performed for the high-resolution structural images. Functional images were further corrected for slice-acquisition time differences, co-registered to the anatomical scan and subsequently spatially normalized to the standard Montreal Neurological Institute (MNI) template. The normalized functional volumes were written out at a 3 × 3 × 3 mm voxel size and were finally smoothed with an 8 mm full-width-at-half-maximum (FWHM) isotropic Gaussian kernel.

On the first level, separate event-related regressors for the EE and SE conditions were included as main regressors of interest. Additionally, separate regressors for the rating phases, the cue phase and the six head motion parameters were included. The regressors were convolved with the standard hemodynamic response function (HRF). To evaluate the sex-dependent effects of OXT on embarrassment an ANOVA including treatment (OXT, PLC), sex (male, female) as between-subject factors and embarrassment type (EE, SE) as a within-subject factor was conducted on the second level.

Based on previous studies indicating that regions involved in arousal (amygdala, anterior insula, AI) and mentalizing (mPFC) neurally underpin embarrassment processing a region-of-interest (ROI) analysis specifically focused on these regions. The amygdala was structurally defined based on masks from the Automated Anatomical Labeling (AAL) atlas (43). Given the size and functional heterogeneity of these regions, the ROIs for the mPFC and anterior insula (specifically dorsal AI, dAI) were defined using 6 mm spheres centered at peak coordinates of these regions reported in a previous study examining the neural basis of embarrassment (30). For the five a priori defined ROIs the first eigenvariate was extracted using Marsbar (44). The individual activity estimates were subsequently subjected to mixed ANOVAs with the between-subject factors treatment (OXT, PLC) and sex (male, female) and the within-subject factor embarrassment type (EE and SE) in SPSS (Statistical Package for the Social Sciences, Version 22). Multiple comparisons for the ROI analysis were controlled for by applying False Discovery Rate (FDR) correction. To explore effects in regions beyond the predefined ROIs an exploratory whole brain analysis was conducted in SPM using cluster-level Family-Wise Error (FWE) correction for multiple comparisons. In line with recent recommendations (45) for the control of false positives using cluster based methods (46) an initial cluster defining threshold of p < .001 (uncorrected) was applied to the data resampled at 3 mm voxel resolution. This voxel-wise analysis employed a flexible factorial design to model the mixed ANOVA computed on the extracted regional estimates and included the within-subject factor embarrassment type (EE and SE) and the between subject factors sex (male, female) and treatment (OXT, PLC). Due to limitations of the flexible factorial design to estimate between-subject main effects, the corresponding main effects of the between-subject factors were additionally examined using separate independent sample *t*-tests and appropriate first level contrasts. Significance level for the corrected *p*-values was *p* < 0.05.

## Results

No significant trait and mood differences were observed between participants in the OXT and PLC group (see Supplementary Table S1).

### Behavioral and SCR results

**Figure 2:**
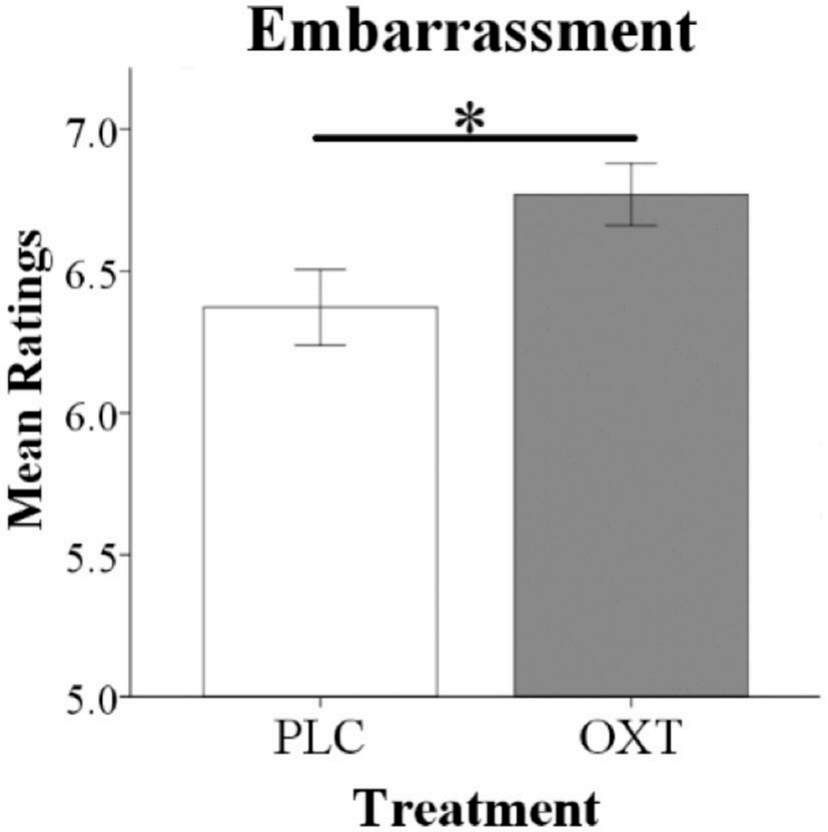
Oxytocin significantly increased overall embarrassment ratings in behavioral tests. Histograms show mean ratings for EE and SE trials combined (^*^p < 0.05).

An ANOVA analysis with treatment and sex as between-subject factors, embarrassment context as a within-subject factor and embarrassment rating as dependent variable, revealed a significant treatment main effect [PLC: 6.45 ± 0.11, OXT: 6.77 ± 0.11, *F*_(1, 66)_ = 4.30, *p* = 0.04, $\eta_{\text{p}}^{2}$ = 0.06] with OXT enhancing embarrassment ratings in both contexts (Figure 2). No significant interaction effects involving treatment were found [Sex ^*^ Treatment, *F*_(1, 66)_ = 2.20, *p* = 0.14, $\eta_{\text{p}}^{2}$ = 0.03; Sex ^*^ Treatment ^*^ embarrassment context, *F*_(1, 66)_ = 0.09, *p* = 0.76, $\eta_{\text{p}}^{2}$ = 0.001].

**Figure 3:**
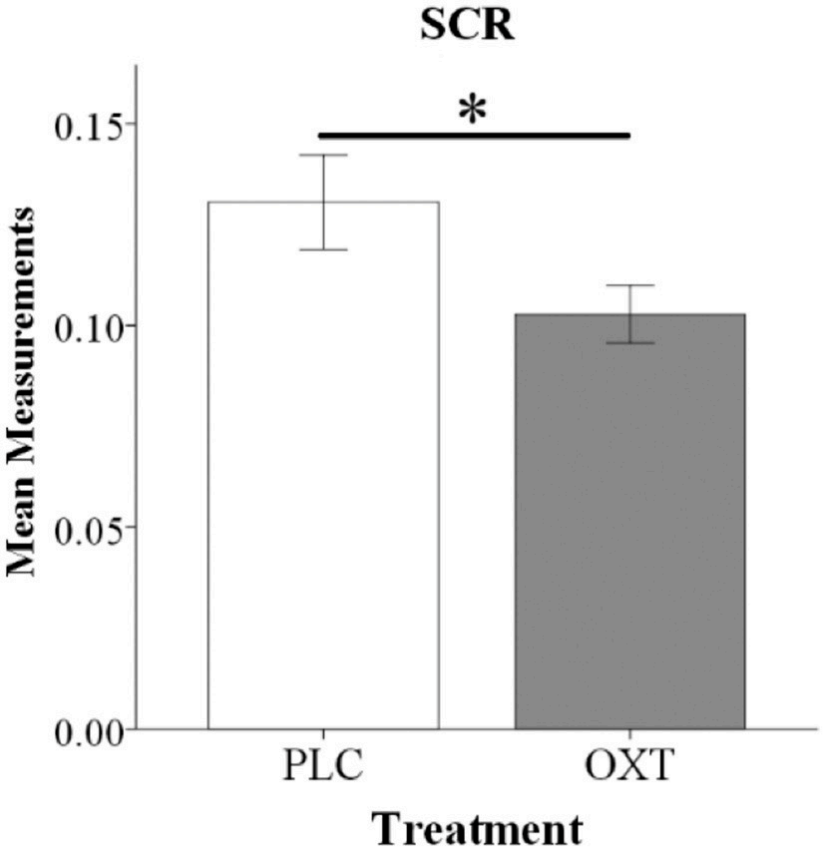
Oxytocin reduced SCR magnitude during the embarrassment task. Histograms show mean SCR magnitude for EE and SE trials combined (^*^P < 0.05).

As a result of scanner-induced noise SCR data from *n* = 12 subjects had to be excluded (baseline readings could not be reliably obtained in >50% of trials) leading to a final sample size of *n* = 58 subjects for the SCR analysis (*n* = 33, OXT; *n* = 25, PLC). An ANOVA analysis including the same factors as for the behavioral analysis and SCR response as dependent variable revealed a significant main effect of treatment [*F*_(1, 54)_ = 7.35, *p* = 0.009, $\eta_{\text{p}}^{2}$ = 0.12] with OXT decreasing the SCR magnitude in both EE and SE conditions (see Figure 3).

### *fMRI* results

**Figure 4:**
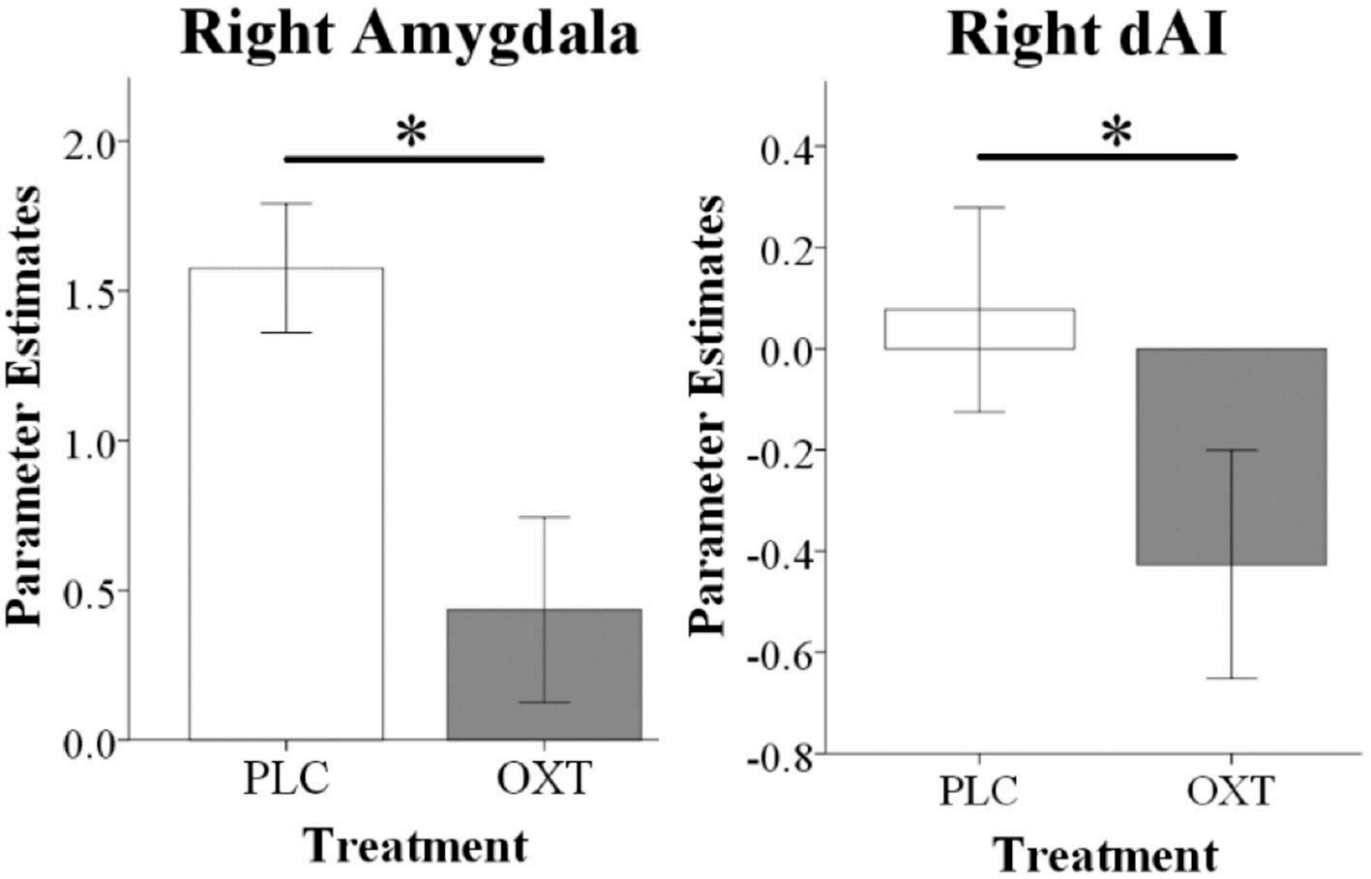
fMRI analysis showing that oxytocin significantly reduced right amygdala and right dAI responses during EE and SE trials. Histograms show parameter estimates for EE and SE trials combined (^*^P < 0.05).

For the ROI-based analysis we initially explored whether there were either effects of embarrassment type or gender in the PLC group. This revealed a main effect of embarrassment type in the mPFC [*F*_(1, 33)_ = 5.54, P_FDR_ = 0.04, $\eta_{\text{p}}^{2}$ = 0.14] and in the amygdala [*F*_(1, 33)_ = 7.21, P_FDR_ = 0.01, $\eta_{\text{p}}^{2}$ = 0.18] but not in the dAI. This confirmed our expectation that there would be a difference in activation in mentalizing in the SE compared to the EE condition and additionally that there was greater activation in the amygdala during EE compared to SE trials. Examination of OXT by comparing the OXT and the PLC treated groups revealed significant main effect of treatment on right amygdala [*F*_(1, 66)_ = 9.97, P_FDR_ = 0.01, $\eta_{\text{p}}^{2}$ = 0.10] and right dAI [*F*_(1, 66)_ = 5.82, P_FDR_ = 0.03, $\eta_{\text{p}}^{2}$ = 0.08] responses, with OXT reducing activity in both EE and SE contexts (see Figure 4). No interactions between treatment and gender and embarrassment type were found in these regions. The exploratory whole-brain analysis did not reveal significant main or interaction effects of treatment on the neural level (P_FWE_ < 0.05).

### Correlation between behavioral, physiological, and neural data and trait questionnaires

**Figure 5:**
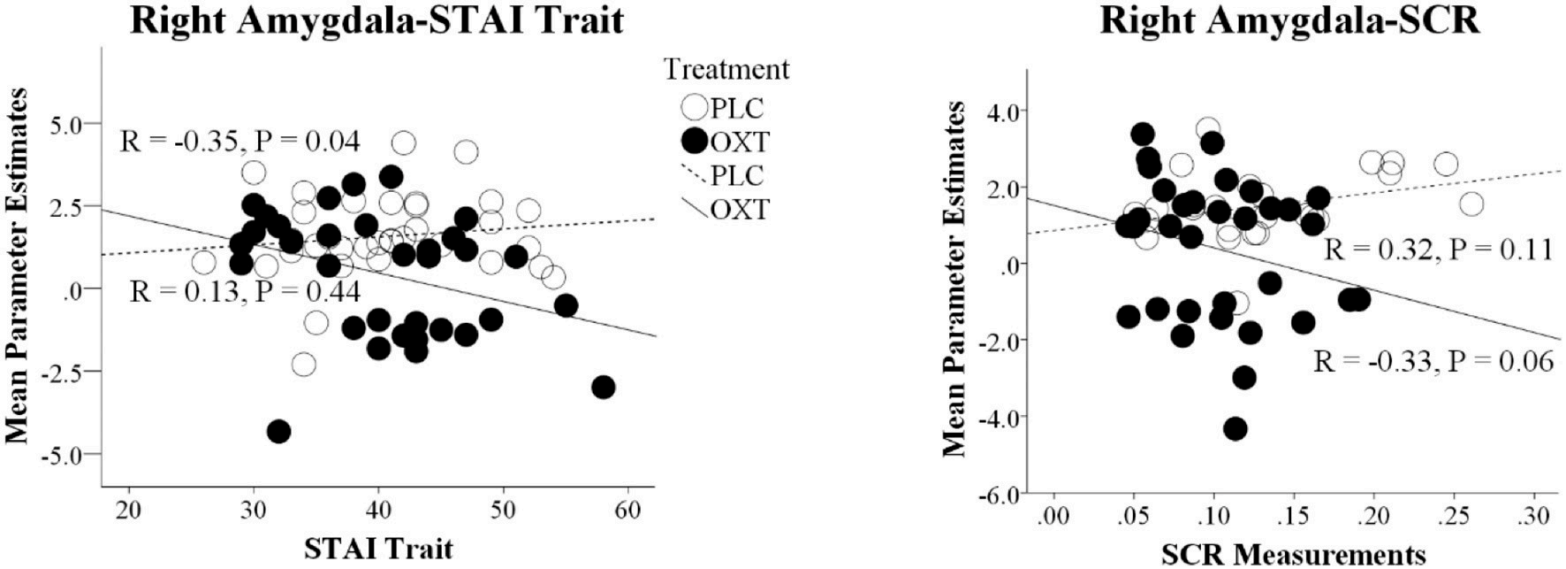
Correlation differences between parameter estimates of right amygdala and trait anxiety scores (STAI Trait) and the magnitude of the skin conductance responses (SCR) in the placebo (PLC) and oxytocin (OXT) groups. Data from EE and SE trials are combined. In both cases the correlation difference between the OXT and PLC groups is significant (Fisher's *Z*-test *p* < 0.05).

There was a negative association between overall embarrassment ratings (average of EE and SE) and STAI Trait scores in the OXT but not the PLC group (STAI Trait: PLC, *r* = 0.06, *p* = 0.75, OXT, *r* = −0.34, *p* = 0.05), suggesting that ratings were particularly increased under OXT in more anxious individuals. However, the correlation difference did not achieve significance (Fisher's *Z*-Test, *z* = 1.66, *p* = 0.098). For the neural data there was a similar negative association between right amygdala activation and STAI trait scores in the OXT group but not the PLC group (STAI Trait: PLC, *r* = 0.13, *p* = 0.44, OXT, *r* = −0.35, *p* = 0.04) and in this case the correlation difference was significant (Fisher's *Z*-Test, *z* = 1.99, *p* = 0.05) (see Figure 5). There was also a negative association between right amygdala activation and the magnitude of the SCR in the OXT group whereas in the PLC group there was a positive association (PLC, *r* = 0.32, *p* = 0.12, OXT, *r* = −0.33, *p* = 0.06; Fisher's *Z*-Test, *z* = 2.40, *p* = 0.016) (see Figure 5). In contrast, no significant associations between levels of autism (ASQ scores) and behavioral, SCR or neural effects were observed (all *ps* > 0.15).

## Discussion

The current experiment demonstrated for the first time that OXT increases both empathic- and self-embarrassment ratings in male and female subjects and that its behavioral effects are associated with decreased responses in the right amygdala and in the right dAI and the magnitude of physiological arousal (SCR). On the other hand, OXT appeared to have no effects on responses in the mentalizing network (mPFC) during either of the embarrassment rating contexts. Furthermore, in contrast with the PLC group the effects of OXT in reducing amygdala responses were negatively correlated with both STAI trait scores and SCR magnitude, implying that it had an anxiolytic effect and particularly in individuals with higher trait anxiety.

Our ROI-based findings in the PLC group that empathic embarrassment primarily increases activation in both mentalizing (mPFC) and emotion processing (amygdala and insula) brain regions is consistent with previous studies (18, 19, 30). Our expectation that the mentalizing component in self- compared to empathic embarrassment would be different was also supported in terms of differential mPFC activation in the PLC group. The effects of OXT in both embarrassment contexts were however restricted to emotion processing regions with reduced amygdala activation, similar to our previous findings for emotional empathy (10) and additionally decreased insula activation. Thus, although a number of studies have reported effects of OXT on both dorsal and ventral mPFC activation in the context of self-vs. other referential and ownership contexts (20, 21) it would appear that this does not occur in self-vs. other-embarrassment. However, these previous studies demonstrating OXT effects on the mPFC in self-vs. other processing have shown that in both behavioral and neural term's it reduced the normal self-bias. Thus, it is possible that OXT had no effects on the mPFC since EE and SE ratings were similar and there was therefore no indication of self-bias for embarrassment experience in the present paradigm.

In contrast to the lack of effects of OXT on self-vs. other processing in the mPFC it increased embarrassment across both, EE and SE in the context of attenuated amygdala and insula reactivity. Although EE trials produced stronger amygdala responses than SE ones following PLC, OXT suppressed them equivalently in both conditions. In line with the social salience hypothesis of OXT (47) the general enhancement of embarrassment may reflect an increased awareness and impact of the social context. However, previous studies reporting OXT-enhanced salience of external social stimuli commonly report decreased amygdala and concomitantly increased insula activity (9, 25, 48) which would be difficult to reconcile with the observed OXT-induced attenuation in both regions in the present study. Decreased insula activity following OXT has been previously observed in response to pain empathy (22), negative social feedback (49) and approach behavior (48). Moreover, in concert with concomitantly decreased amygdala activity attenuated stress in response to negative interactions (50) and reduced anxiety during social sharing (in women) have been reported (51). In line with these previous findings the suppression of both regions may thus rather reflect the stress-buffering or anxiolytic effects of OXT. Together with the hippocampus, the amygdala and insula are considered core nodes of the threat and anxiety circuitry (52, 53) and are hyperactive across anxiety disorders (52)–particularly social anxiety disorder (54). Moreover, successful non-pharmacological therapy for exaggerated anxiety has been accompanied by decreased hyperactivity in these regions (55, 56). The amygdala is also critically involvemed in emotional modulation of the SCR (57) and during OXT-enhanced empathy and protective responses suppression of amygdala reactivity was accompanied by increased SCR (10) and acoustic startle response (26). On the other hand, OXT decreased the SCR in response toward conditioned threat stimuli (58) and decreased autonomic stress reactivity (59). Moreover, increased activity in the dAI has been specifically associated with increased arousal during embarrassing social situations (30), however not embarrassment-related schadenfreude (60). Together, the pattern of decreased reactivity in the two threat nodes as well as the autonomic arousal measures during exposure to embarrassing, social-stressful situations may thus reflect anxiolytic effects of OXT. This interpretation is further supported by a negative association between amygdala activation and trait anxiety scores and the magnitude of the SCR in the OXT group suggesting that it reduced anxiety and particularly in more anxious individuals.

It seems paradoxical therefore that in the context or empathic and self-embarrassment OXT-reduced amygdala responses were associated with a decreased SCR and yet ratings were still increased. This demonstrates that OXT-induced reduction of amygdala activity per se is not sufficient to infer anxiolytic effects of OXT, and indeed another study has also reported that OXT can promote an anxiogenic response in the context of concomitantly reduced amygdala activation (25). It thus may be hypothesized that the insula plays an important role in shaping the specific behavioral effects of an unspecific OXT-attenuation of the amygdala. Whereas concomitantly increased insula activity may promote the salience-enhancing effects of OXT (25, 26, 48), a concomitant reduction may reflect anxiogenic or arousal reducing effects of OXT (22, 48, 49) which is in line with the contribution of the anterior insula to several functional domains including salience, and arousal (61, 62). The observed pattern of neural effects of OXT in the present study may thus favor an anxiolytic interpretation of its action during embarrassment processing. This may also reflect a reduction of the aversive and threatening affect evoked by the embarrassment stimuli and facilitate a more cognitive appraisal of the social contexts and their implications thus enhancing embarrassment levels across situations experienced both by others or by self.

Thus, OXT may have reduced the feelings of both pain and anxiety experienced during embarrassment, leading to a more cognitive assessment such that subjects rated the level of embarrassment experienced as higher. In this respect it should be noted that subjects in our task were asked to rate the level of embarrassment being experienced in a particular situation and not specifically to rate the intensity of their feelings for others in embarrassing situations, or of their own personal feelings in the self-condition. It is possible therefore that in the context of moral self-conscious emotions such as embarrassment, OXT may actually reduce emotional empathy toward others in order to promote a more accurate cognitive assessment of what someone is feeling. This might in turn promote more efficient avoidance of potential embarrassing situations in the future. Further experiments are required to disentangle these cognitive and emotional factors.

In our previous experiment using the MET task we observed that the OXT enhancement of emotional empathy ratings and reduced amygdala activity showed some associations with trait autism, however in the current study we did not find this. Previous studies have reported decreased responses in the anterior insula to social pain in adults with autism spectrum disorder (19), although not for empathic neural responses toward physical pain (63). However, we found no associations between neural responses to embarrassment and autistic traits in healthy subjects. On the other hand, we did find a negative association between trait anxiety and the effect of OXT on the amygdala and SCR, which contrasted with no such association in the PLC group. This suggested that OXT was particularly attenuating neural and behavioral indices of anxious arousal in subjects with high trait anxiety, and resonates with previous findings suggesting that OXT particularly reduced negative appraisal following social stress induction in high trait anxiety subjects (36). These observations conflict with a recent report on OXT-induced increased startle responsivity in high anxious subjects (38). However, in this previous study OXT specifically increased startle responsivity toward non-social stimuli, further emphasizing how complex interactions between personal and social contextual factors may moderate the specific effects of OXT.

No sex-differential effects were observed in the behavioral effects of OXT on embarrassment ratings or on right amygdala or right insula responses, which is consistent with our previous observations that OXT enhanced emotional empathy in the MET in both sexes (10). These findings contrast with other studies reporting sex-dependent responses in amygdala and insula (23, 64) in the context of the impact of positive and negative personal characteristics on face attraction and sub-liminal processing of emotional faces. However, this may reflect the fact that exhibiting empathic-embarrassment and self-embarrassment are of equal adaptive importance in males and females and that OXT only tends to promote or amplify sex-differences in behavioral and neural responses associated with sex-specific priorities in social salience and social preference processing (see 22).

It is still unclear how intranasal administration of OXT exerts its neural and behavioral effects. While it has been questioned whether OXT administered via an intranasal spray does actually enter the cerebrospinal fluid (CSF) and influence brain OXT receptors (65) it is now established from work using labeled peptides in monkeys that it does enter into the CSF as well as the blood (66). In humans intranasal OXT does also increase CSF as well as blood OXT concentrations although with different time courses (67). It has still to be demonstrated however whether it can both enter the brain across the blood brain barrier from increased concentrations in the peripheral vasculature (66) and directly via the established lymphatic or other transport mechanisms at the back of the nasal cavity [see (8)]. Importantly however, despite some early studies in autistic subjects reporting behavioral effects of intravenous oxytocin (68, 69) recent studies have failed to find any behavioral or neural effects of intravenous compared with intranasal routes of administration (70, 71). Intranasal administration of OXT in humans also results in extensive increases in regional cerebral blood flow in the majority of brain regions containing OXT receptors at the same time course as routinely used in the current as well as most previous intranasal treatment studies (i.e., around 45 min)(72). Thus, in the context of the findings in the current experiment it is likely that functional effects of OXT on embarrassment and reduced physiological arousal, as well as those on the amygdala and insula, are caused primarily by its actions following entry into the brain, although we cannot rule out some contributions from indirect actions via receptors in peripheral organs.

Findings of the present study need to be interpreted in the context of limitations. Firstly, compared to previous studies that determined sex-differential neural effects of OXT during evaluation social-emotional stimuli the present sample was smaller [*n* = 70, previous studies enrolled slightly higher samples between around *n* = 80–90 subjects, (23, 64)]. The lack of sex-differential effects of OXT on embarrassment therefore needs to be replicated in larger samples. Secondly, our task did not adequately separate cognitive from emotional components of embarrassment in order to provide a better understanding of how OXT attenuation of both amygdala and insula responses resulted in increased ratings of embarrassment.

Overall therefore we have demonstrated for the first time that OXT can enhance ratings of both empathic and self-embarrassment in males and females, showing that it also influences moral, self-conscious emotional responses. These behavioral effects of OXT are associated with decreased physiological arousal and decreased responses in both the right amygdala and anterior insula, but not in mentalizing networks (mPFC). Furthermore, OXT effects on the amygdala are strongest in individuals with high trait anxiety. Thus, OXT in this context may be promoting an anxiolytic effect resulting in a more cognitive rather than emotional appraisal of embarrassment levels.

## Author contributions

YG and KK designed this experiment, YG collected the data, YG, WZ, FZ, KK, and BB analyzed the data, YG, WZ, XM, SY, BB, and KK interpreted the results. YG, BB, and KK wrote the paper.

### Conflict of interest statement

The authors declare that the research was conducted in the absence of any commercial or financial relationships that could be construed as a potential conflict of interest.
