# Supplementary material for: Oxytocin Facilitates Empathic- and Self-embarrassment Ratings by Attenuating Amygdala and Anterior Insula Responses
Source: Front Endocrinol (Lausanne). 2018 Sep 25;9:572. doi: 10.3389/fendo.2018.00572 (PMC6190868; doi:10.3389/fendo.2018.00572)
Supplement: Supplementary file 1 [file Table_1.DOCX]

| Measurements | PLC | OXT | T-value | P |
| --- | --- | --- | --- | --- |
| Age (yr) | 21.86 ± 0.33 | 22.03 ± 0.36 | -0.35 | 0.73 |
| Education | 15.89 ± 0.28 | 16.09 ± 0.28 | -0.51 | 0.61 |
| Empathy Quotient (EQ) | 38.17 ± 1.72 | 37.83 ± 1.36 | 0.16 | 0.88 |
| Beck Depression Inventory (BDI) | 7.43 ± 1.05 | 4.91 ± 0.89 | 1.83 | 0.07 |
| State and Trait Anxiety Inventory (STAI)_State | 39.74 ± 1.54 | 37.94 ± 1.14 | 0.94 | 0.35 |
| State and Trait Anxiety Inventory (STAI)_Trait | 40.63 ± 1.19 | 40.37 ± 1.25 | 0.15 | 0.88 |
| Positive and Negative Affect Scale (PANAS)_Positive | 29.97 ± 0.99 | 29.17 ± 0.88 | 0.61 | 0.55 |
| Positive and Negative Affect Scale (PANAS)_Negative | 19.91 ± 1.16 | 18.37 ± 0.90 | 1.05 | 0.30 |
| The Adult Autism Spectrum Quotient (ASQ) | 20.14 ± 0.98 | 19.06 ± 1.13 | 0.72 | 0.47 |
| Wong Law Emotional Intelligence Scale-Chinese(WLEIS-C) | 83.71 ± 1.78 | 83.00 ± 2.33 | 0.24 | 0.81 |
| Liebowitz Social Anxiety Scale (LSAS)_Fear | 43.66 ± 1.82 | 42.74 ± 1.77 | 0.36 | 0.72 |
| Liebowitz Social Anxiety Scale (LSAS)_Avoidance | 42.09 ± 1.87 | 40.83 ± 1.86 | 0.48 | 0.64 |
| The Self-Esteem Scale (SES) | 31.71 ± 0.63 | 32.60 ± 0.67 | -0.96 | 0.34 |

**Table S1** Demographic and psychometric characterization of the treatment groups
